# A Collective Route to Head and Neck Cancer Metastasis

**DOI:** 10.1038/s41598-017-19117-9

**Published:** 2018-01-15

**Authors:** Arutha Kulasinghe, Henri Schmidt, Chris Perry, Bernard Whitfield, Liz Kenny, Colleen Nelson, Majid E. Warkiani, Chamindie Punyadeera

**Affiliations:** 1 The School of Biomedical Sciences, Institute of Health and Biomedical Innovation, Queensland University of Technology, Kelvin Grove, QLD Australia; 2 Department of Otolaryngology, Princess Alexandra Hospital, Woolloongabba, QLD Australia; 3 School of Medicine, University of Queensland, Royal Brisbane and Women’s Hospital, Brisbane, Australia; 4 Central Integrated Regional Cancer Service, Queensland Health, Brisbane, Queensland Australia; 5 Australian Prostate Cancer Research Centre - Queensland, Institute of Health and Biomedical Innovation, Queensland University of Technology, Princess Alexandra Hospital, Woolloongabba, QLD Australia; 6 The School of Biomedical Engineering, University of Technology, Sydney, NSW Australia; 7 Tranlsational Research Institute, Brisbane, Australia

## Abstract

Distant metastasis (DM) from head and neck cancers (HNC) portends a poor patient prognosis. Despite its important biological role, little is known about the cells which seed these DM. Circulating tumour cells (CTCs) represent a transient cancer cell population, which circulate in HNC patients’ peripheral blood and seed at distant sites. Capture and analysis of CTCs offers insights into tumour metastasis and can facilitate treatment strategies. Whilst the data on singular CTCs have shown clinical significance, the role of CTC clusters in metastasis remains limited. In this pilot study, we assessed 60 treatment naïve HNC patients for CTCs with disease ranging from early to advanced stages, for CTC clusters utilizing spiral CTC enrichment technology. Single CTCs were isolated in 18/60–30% (Ranging from Stage I-IV), CTC clusters in 15/60–25% (exclusively Stage IV) with 3/15–20% of CTC clusters also containing leukocytes. The presence of CTC clusters associated with the development of distant metastatic disease(*P* = 0.0313). This study demonstrates that CTC clusters are found in locally advanced patients, and this may be an important prognostic marker. *In vivo* and *in vitro* studies are warranted to determine the role of these CTC clusters, in particular, whether leukocyte involvement in CTC clusters has clinical relevance.

## Introduction

Head and neck cancers (HNC) accounts for the fifth most common non-skin cancer globally^1^. Despite the fact that primary treatment is usually both intensive and highly morbid, up to 50% of HNC patients still fail locoregionally or systemically. The more advanced the locoregional disease, the greater the risk of presenting with established metastatic disease, or harbouring micrometastatic disease, resulting in systemic failure at a later point. Circulating tumour cells (CTCs), which are shed from primary or secondary tumours and circulate in patients’ blood represent an important window into the mechanisms and characteristics of tumour metastasis^2–4^. They may also help direct locoregional and systemic treatment, by stratifying patients’ risk of systemic failure, allowing better selection of treatment individually.

CTCs were first described by Thomas Ashworth in 1869, as ‘cells identical with those of the cancer itself’, and the field has rapidly advanced in the last decade^5–8^. CTCs can be measured non-invasively from the blood and have direct clinical application^9^. In 2004, the FDA approved the first CTC enumeration platform, CellSearch (Janssen Diagnostics)^10,11^. It was demonstrated on this platform that single CTCs, in a number of tumour types, had clinical utility^10^. The enumeration of CTCs and cut off values of 5 or more CTCs in 7.5 ml of blood for metastatic breast cancer and prostate cancer has been associated with a poor prognosis and predictive of shorter progression free survival (PFS) and overall survival (OS)^2,12^. However, due to its inherent pre-selection of epithelial tumour cells expressing EpCAM, this system has shown poor sensitivity in detecting CTCs isolated from HNC patients bloods^13,14^.

Of recent, there has been a shift, from marker-based CTC assays to marker-independent assays, to capture a greater population of CTCs from circulation, including CTC clusters^2,15–17^. In a number of studies, CTC clusters or circulating tumour microemboli (CTM), composed of platelets, stromal and hematopoietic cells, have been reported^13,16,18^. Critically, these tumour cell aggregates, are protected from the shear stressors in the blood, and are better suited to survive the journey through the circulation by cooperation of heterogeneous cell types within the cluster, which can include immune evading cells^2,17,19^. The prevalence and the number of CTC clusters can be underestimated due to their short detection window and lack of appropriate detection methods^20^. Studies have also documented that CTC clusters have a shorter circulation half-life, with faster entrapment within distant organs where metastatic growth may initiate^10,19,21,22^. CTC clusters may provide clues to their evolution during the course of cancer treatment and the mechanisms of cluster mediated treatment resistance^23^. CTC clusters have been associated with decreased metastasis-free survival and a greater metastatic capacity than single CTCs^16,17,21^. Notably, it has been shown that CTC clusters, held by plakoglobin-dependent adhesions, have arisen from oligoclonal expansion of tumour cell groupings, rather than aggregation or proliferation of single CTCs^17^. Recent studies suggest that CTC clusters have the ability to traverse narrow capillaries in a ‘single-file’ and retain the ability to re-form the intact cluster upon exiting, highlighting the metastatic seeding capacity of these large cellular aggregates that were previously thought to extravasate upon reaching narrow capillaries^16,24^.

To date, the role of CTC clusters, including CTM, has not been well established. Despite their biological importance, in comparison to single CTCs, the data on CTC clusters remains unclear. In this pilot study, we investigated whether CTC clusters and CTM were found in the circulation of HNC patients. This study evaluated 60 treatment-naive HNC patients, with disease ranging from early to advanced stages, for CTC clusters using spiral microfluidic CTC enrichment technology. This technology enriches for CTCs by a function of cell size and deformability and provides a robust methodology for CTC enrichment^15,25,26^.

## Results

**Figure 1:**
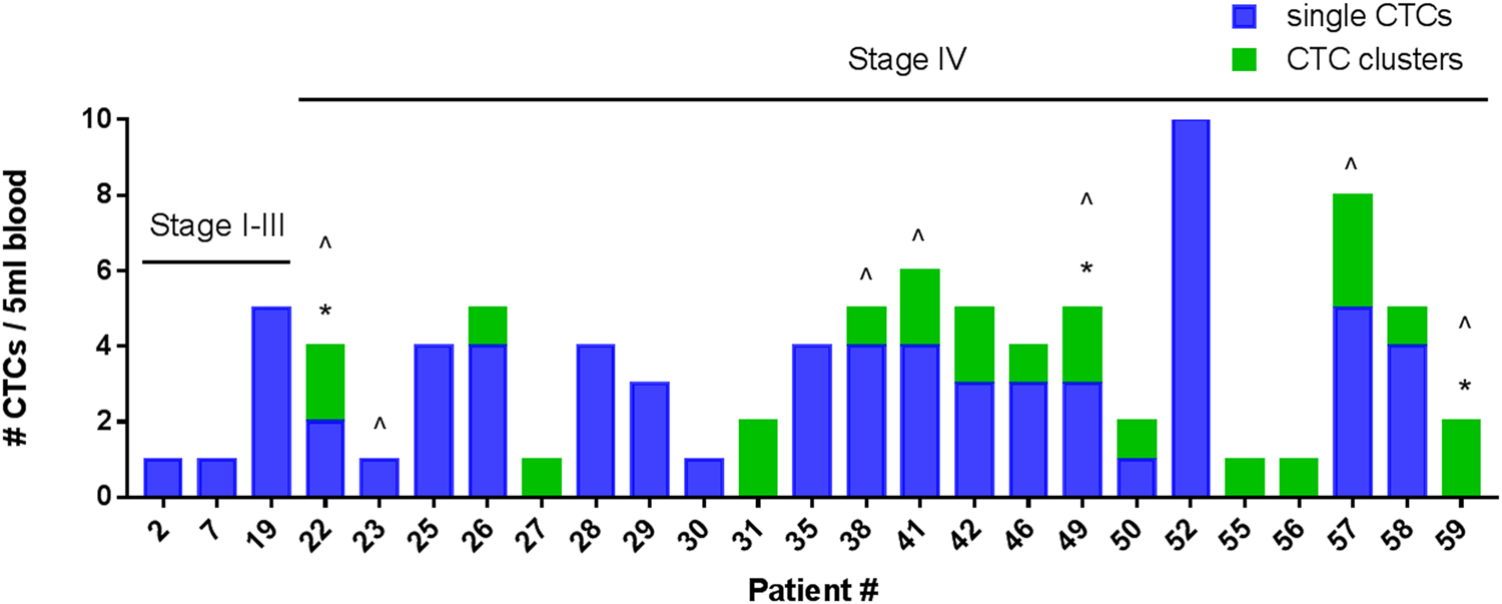
Stacked bar graph showing single CTCs (blue), CTC clusters (green) for each of the 25 head and neck cancer patients positive for either/both cell types from 5 ml blood draw. CTC positive: 3/22 Stage I-III, 22/39 Stage IV patients). *Asterix* (*) *represent the CTC clusters with white blood cell involvement*. *Caret* (^) *refers to patients that developed lung or liver lesions within 3–6 month period*.

Patients’ HNC disease staging ranged from early to advanced stages of HNC (Stage I-IV). CTCs and CTC clusters were successfully isolated using the spiral microfluidic chip. All patients had no evidence of distant metastatic disease upon presentation, however, at later time points (3–6 months follow up rescan), 7 stage IV patients developed lung and/or liver lesions (Fig. 1). CTC clusters were present in the blood of 6/7 patients that progressed to have distant disease. The patient demographics and clinicopathological features are presented in Table 1 and Table 2 respectively.

**Table 1:** Patient demographics (n = 60).

Variables	N
Total	60 (100%)
**Gender**	
Male	53 (88.3%)
Female	7 (11.7%)
***Age (years)***	
≤60	25
>60	35
***Anatomic site of primary***	
Oral Cavity	24
Oropharyngeal	27
Larynx	5
Hypophaynx	3
Salivary Glands	1
***Tumour Staging***	
I	6
II	6
III	9
IV	39
***Distant metastases***	
M0	60
M1	0
***HPV status***	
HPV-positive	26
HPV-negative	29
HPV status unknown	5
***CTC status***	
CTC-positive (single cells)	20/60 (33.3%) (Range from 1–10CTCs/5 ml blood)
CTC-positive (clusters)	15/60 (25%) (Range from 1–3/5 ml blood)
CTC clusters including WBCs	3/15 (20%)

*WBCs: White blood cells.

**Table 2:** Clinicopathological findings (n = 60).

Pt #	Gender	Age	HPV status	Staging	Site	Single CTCs	CTC clusters	# cells per cluster	Follow up FDG Pet Scan
1	f	58	negative	I	Oral Cavity	0	0	0	
2	m	57	negative	I	Oral Cavity	1	0	0	
3	m	69	negative	I	Oropharynx	0	0	0	
4	m	57	negative	I	Oral Cavity	0	0	0	
5	m	71	negative	I	Oral Cavity	0	0	0	
6	f	64	unknown	I	Oral Cavity	0	0	0	
7	m	78	negative	II	Larynx	1	0	0	
8	m	55	negative	II	Oral Cavity	0	0	0	
9	m	62	negative	II	Oral Cavity	0	0	0	
10	m	59	positive	II	Oral Cavity	0	0	0	
11	m	77	negative	II	Oral Cavity	0	0	0	
12	m	64	negative	II	Oral Cavity	0	0	0	
13	m	56	negative	III	Oral Cavity	0	0	0	
14	m	55	negative	III	Oral Cavity	0	0	0	
15	m	69	negative	III	Larynx	0	0	0	
16	m	63	negative	III	Oropharynx	0	0	0	
17	m	63	negative	III	Oropharynx	0	0	0	
18	m	78	negative	III	Oral Cavity	0	0	0	
19	m	66	unknown	III	Larynx	5	0	0	
20	f	62	positive	III	Oral Cavity	0	0	0	
21	m	63	unknown	III	Larynx	0	0	0	
22	m	74	positive	IV	Oral Cavity	2	2*	13,8	lung lesion
23	m	88	negative	IV	Oral Cavity	1	0	0	lung lesion
24	m	81	positive	IV	Oropharynx	0	0	0	
25	m	60	Positive	IV	Oropharynx	4	0	0	
26	m	64	negative	IV	Oral Cavity	4	1	6	
27	m	50	Positive	IV	Oropharynx	0	1	3	
28	f	65	negative	IV	Oral Cavity	4	0	0	
29	m	74	positive	IV	Oropharynx	3	0	0	
30	f	45	positive	IV	Oropharynx	1	0	0	
31	m	58	Positive	IV	Oropharynx	0	2	3,3	
32	m	73	negative	IV	Oropharynx	0	0	0	
33	m	73	negative	IV	Oral Cavity	0	0	0	
34	m	55	positive	IV	oropharynx	0	0	0	
35	m	56	negative	IV	Oropharynx	4	0	0	
36	m	69	positive	IV	Oropharynx	0	0	0	
37	m	62	positive	IV	Oropharynx	0	0	0	
38	m	58	positive	IV	Oropharynx	4	1	5	lung lesion
39	m	66	positive	IV	Oropharynx	0	0	0	
40	m	79	positive	IV	Oropharynx	0	0	0	
41	m	66	negative	IV	Larynx	4	2	8,10	lung and liver lesions
42	m	50	positive	IV	Oral Cavity	3	2	3,5	
43	m	75	positive	IV	Oral Cavity	0	0	0	
44	m	54	positive	IV	Oropharynx	0	0	0	
45	f	70	positive	IV	Oropharynx	0	0	0	
46	m	53	negative	IV	Oropharynx	3	1	6	
47	m	77	negative	IV	Hypophaynx	0	0	0	
48	m	50	unknown	IV	Oral Cavity	0	0	0	
49	m	65	negative	IV	Oropharynx	3	2*	5,7	liver lesion
50	m	58	unknown	IV	Oropharynx	1	1	3	
51	m	74	positive	IV	Oropharynx	0	0	0	
52	m	62	positive	IV	Oropharynx	10	0	0	
53	m	23	negative	IV	Hypopharynx	0	0	0	
54	m	61	positive	IV	Hypopharynx	0	0	0	
55	m	50	positive	IV	Oral Cavity	0	1	3	
56	f	59	negative	IV	Oral Cavity	0	1	4	
57	m	59	positive	IV	Oropharynx	5	3	5,5,7	lung lesion
58	m	50	positive	IV	Oropharynx	4	1	3	
59	m	64	positive	IV	Oropharynx	0	2*	7,5	lung lesion
60	m	60	negative	IV	Salivary Glands	0	0	0	

*CTC clusters with WBCs.

### Single CTCs

**Figure 2:**
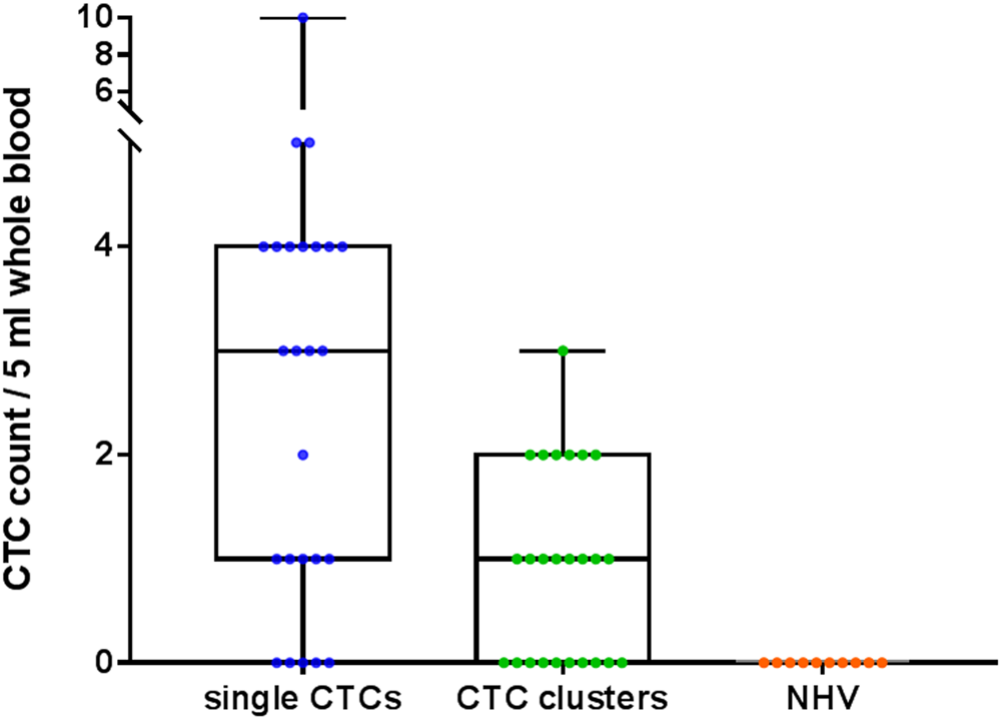
Box and whisker plot showing the number of single CTCs (blue) and CTC clusters (green) (pan-CK + EGFR+ DAPI +) per 5 ml blood for the 25 CTC positive head and neck patient samples and 10 normal healthy volunteers. The box and represent the minimum to maximum values with all individual data points.

Single CTCs were detected in 20/60 patients (33.3%) ranging from 1–10 CTCs/5 ml (Fig. 2) (CTC positive: 1/6 Stage I, 1/6 Stage II, 1/9 Stage III, 17/39 Stage IV).

### CTC Clusters

**Figure 3:**
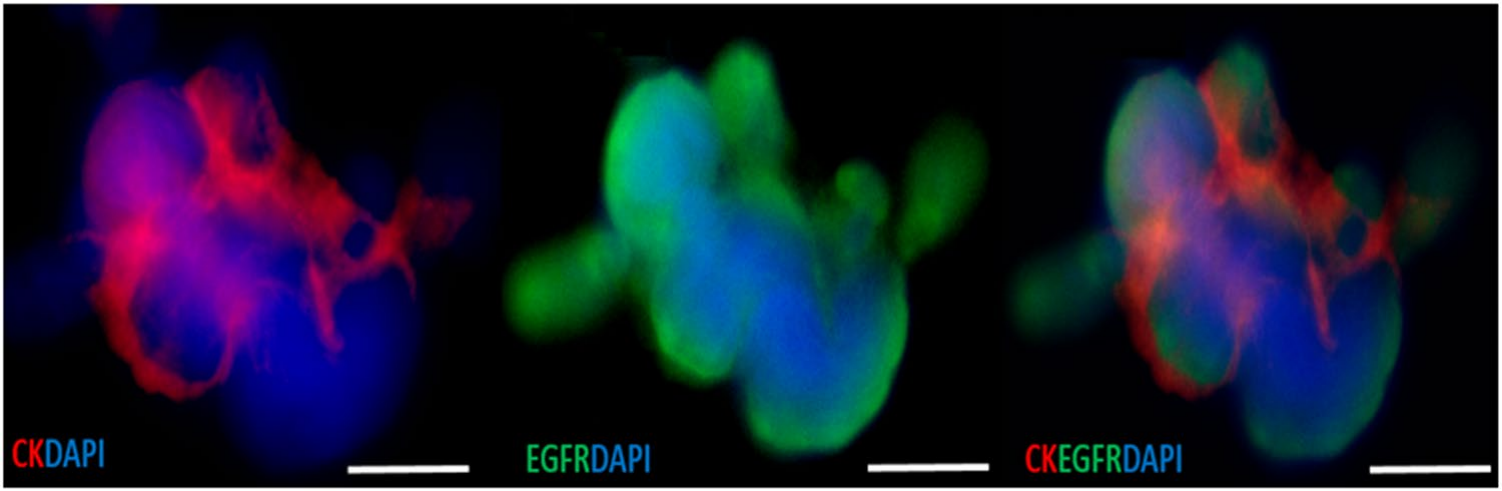
Circulating tumour cell (CTC) clusters isolated from a head and neck cancer (HNC) patient stained positive for pan-cytokeratin −8, 18, 19 (Red), EGFR (Green), DAPI (Blue) and negative for CD45 (not shown). Scale bar represents 10 µm.

**Figure 4:**
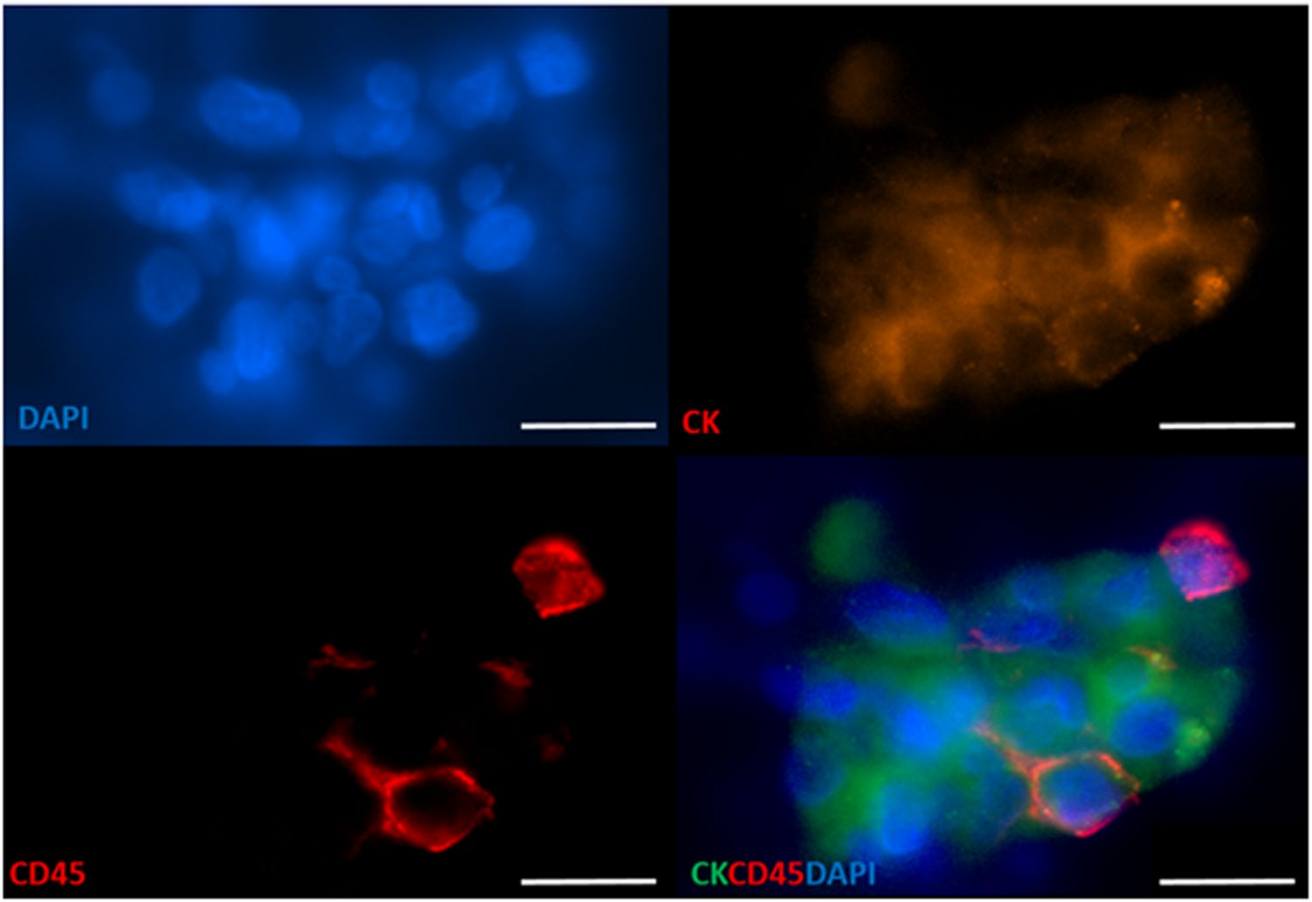
Circulating tumour cluster (pan-cytokeratin + EGFR + DAPI+), containing white blood cells (CD45 + DAPI+ ) within the cellular aggregate. Scale bar represents 10 µm.

CTC clusters were detected in 15/60 patients (25%) ranging from 1–3 clusters/5 ml (consisting of 3–13 cells) (Figs 2 and 3) (CTC positive: 0/6 Stage I, 0/6 Stage II, 0/9 Stage III, 15/39 Stage IV), with white blood cells present in 3/15 CTC clusters (20%) (Fig. 4).

In 10/20 patients positive for single CTCs, CTC clusters were not present. Whereas, in 5/15 patients positive for CTC clusters, single CTCs were not found. Patients presented with both single and CTC clusters in 10/60 patient samples. No CTC-like (single/clusters) events were observed in the 10 normal healthy volunteer (NHV) cohort.

## Discussion

Single CTCs have been previously reported in other solid tumour types including HNC^5,6,25,27–29^. CTC clusters are defined as ≥3 tumour cells, held in close proximity by strong cell-cell adhesions, detected in the blood of cancer patients^2,30,31^. Studies have reported that CTC clusters have a shorter half-life in blood, an increased metastatic capacity compared to single CTCs^21,24,30^, and that disrupting the interactions within clusters may provide a strategy to reduce CTC cluster mediated metastasis^16^. An example of this would be the knockdown of plakoglobin, a protein which is highly expressed in CTC clusters that may facilitate in the reduction of cluster generation^21,32^. The observation of CTC clusters or CTMs has been associated with adverse outcomes^33^.

To date, the clinical utility of CTCs in HNC remains limited, and few studies have reported on the presence of these subpopulations of CTC clusters^2,13,14,34–37^. In our cross sectional pilot study, single CTCs were found in 33.3% of patients (Stage I-IV) and CTC clusters in 25% of patients (Stage IV). Whilst the number of single CTCs is comparable to previous HNC studies^2,14,26,34,38–41^, the presence of CTC clusters in 25% of the cohort is of importance^42,43^. Furthermore, from 7 stage IV HNC patients in the study, that progressed to developing lung/liver metastasis (3–6 months later), 6 patients presented with CTC clusters (*P* = 0.0313). In patients with the absence of CTC clusters, the predicitive value of not developing distant disease within 6 months was found to be 95%. Chalmers *et al*., 2012 has shown that HNC CTC clusters can co-express Vimentin and CD44, epithelial-mesenchymal transition (EMT) and stemness traits which may represent an aggressive phenotype^34^. It is not fully understood whether the expression of mesenchymal traits on CTC clusters is due to single proliferating CTCs which had undergone EMT or an EMT transformed CTC cluster^44^. Single CTCs, CTC clusters, or both cell types were found in 25/60 of the sampled HNC patient bloods. Importantly, the presence of CTC clusters did not depend on single CTCs being present and could be potentially used as an independent prognostic marker^44^.

Notably, in 3/21 of the CTC clusters from stage IV HNC patients, white blood cells were found within the cluster. This feature in a number of CTC clusters is of importance as the incorporation of white blood cells (WBCs) in the CTC cluster may provide a mechanism by which these CTC clusters evade the immune system^31,45–47^. To this end, recent studies have highlighted that PD-L1 is frequently expressed on CTCs and may be involved in immune evasion^45,46,48^. Studies have shown that the presence of non tumour cells (e.g. platelets and leukocytes) within CTC clusters promotes metastasis, by protecting the clusters from the shear stressors and immune attacks^44,49^.

## Conclusion

This pilot study challenges the notion of only reporting on individual CTCs in HNC studies. Whilst single CTCs were found in the screened population, a comparable population presented with CTC clusters. Whilst the role of CTC clusters, including clusters containing WBCs is not fully understood, studies into this area are warranted to understand cluster mediated immune escape and their role in metastasis.

## Materials and Methods

### Study design

This prospective study was conducted across three major academic hospitals in Brisbane, Austrlia. Ethics approval was obtained from the Metro South and Health Service District Human Research Ethics Committee (HREC/12/QPAH/381 and HREC/11/QPAH/331) in accordance with the National Health and Medical Research Council’s (NHMRC) guidelines to collect blood from the Royal Brisbane and Women’s Hospital (RBWH), Logan Hospital and Princess Alexandra Hospital (PAH). This study also has QUT ethics approval (1400000617 and 1100001420). All participants gave written informed consent and 10 ml blood samples were collected in BD Vacutainer K2E tubes (EDTA) from 60 HNC patients before treatment and 10 normal healthy volunteers (NHV), with CTC assessment made as described below.

### Enrichment of CTCs using spiral technology

An initial red blood cell (RBC) lysis (Astral Scientifix) was performed to the 10 ml blood sample to reduce the cellular components passing through the spiral chip. Thereafter, cells were centrifuged and the pellet resuspended in 10 ml of sheath buffer (1xPBS, 2 mM EDTA, 0.5% BSA). The spiral device was setup as previously described^15,26^. In brief, after the spiral chip had an initial priming run, the sample was loaded onto a syringe and pumped through the spiral chip at 1.7 ml/min. The CTC output were collected and spun down at 300 × g for 5 mins.

### CTC and CTC cluster characterization

CTC enriched cells were cytospun onto glass slides and CTCs/CTM identified using the CellSearch antibody cocktail (Cytokeratin-8,18,19, CD45, DAPI) (Janssen Diagnostics). Cells were further characterized for surface EGFR using anti-EGFR antibody (AY13, Biolegend, San Diego). Briefly, the glass slides were incubated with an antibody cocktail of CellSearch Reagents (20 µl staining reagent, 20 µl permeabilization buffer, 20 µl fixation buffer, 10 µl DAPI in 120 µl PBS) for 1 hour at room temperature, washed 3 times in PBS, coverslipped and imaged on the Olympus IX73 epifluorescence microscope.

### CTC and CTM parameters

CTCs were visualized using immunofluorescence post enrichment. Cells were classified as CTCs after meeting the following criteria (i) high nucleus to cytoplasmic ratio (ii) morphologically larger than the background cells with intact nuclei (iii) cytokeratin-8,18,19 positive (iv) EGFR positive (v) CD45 negative. CTC clusters were reported as 3 or more CTCs in close proximity and CTM when CTC clusters included leukocytes (CD45 positive cells). The results were reported as the number of CTCs, CTC clusters, and CTM per 5 ml whole blood.

### Statistical Analysis

The development of distant metastatic disease (confirmed by imaging and biopsy where possible) were compared to CTC groups using Fisher’s exact test. All statistical analysis were performed using Graphpad Prism 7.0 software, were two-sided, and *P*-values <0.05 considered statistically significant.

### Data Availability

All data generated or analysed during this study are included in this published article.
